# *Aspidoras mephisto*, new species: The first troglobitic Callichthyidae (Teleostei: Siluriformes) from South America

**DOI:** 10.1371/journal.pone.0171309

**Published:** 2017-03-01

**Authors:** Luiz Fernando Caserta Tencatt, Maria Elina Bichuette

**Affiliations:** 1 Departamento de Biologia, Núcleo de Pesquisas em Limnologia, Ictiologia e Aquicultura, Programa de Pós-Graduação em Ecologia de Ambientes Aquáticos Continentais, Universidade Estadual de Maringá, Maringá, Paraná, Brazil; 2 Departamento de Ecologia e Biologia Evolutiva, Laboratório de Estudos Subterrâneos, Universidade Federal de São Carlos, São Carlos, São Paulo, Brazil; Universita degli Studi di Roma La Sapienza, ITALY

## Abstract

*Aspidoras mephisto* n. sp. is described from the Anésio-Russão cave system, upper Tocantins River basin, Goiás, Brazil. The species can be readily distinguished from its congeners by troglomorphic features and also by presenting the following combination of features: infraorbital 1 generally with well-developed ventral laminar; or moderately developed; poorly-developed serrations on posterior margin of pectoral spine; nuchal plate not externally visible; dorsal fin, even in conspicuously colored specimens, with only dark brown or black chromatophores concentrated on rays, forming spots in some specimens; membranes hyaline; or sparse dark brown or black chromatophores on membranes, not forming any conspicuous pattern; and inner laminar expansion of infraorbital 1 moderately developed. Information about its habitat, ecology, behaviour and conservation status are provided and also a brief description of the juvenile stage.

## Introduction

The Callichthyidae are small- to medium sized armored catfishes, which can be promptly recognized by the presence of two longitudinal series of dermal plates on flanks [1]. The family currently presents around 200 valid species, eight genera, and is widely distributed throughout South America, occurring in all of its cis-Andean portion and also in trans-Andean Colombia and Panama [1, 2]. Corydoradinae Hoedeman currently harbors *Aspidoras*, *Corydoras* and *Scleromystax*, and can be recognized by the combination of several features, such as: narrow frontal bones, lanceolate genital papilla in males, dorsal hypohyal present and hypobranchial 1 deep [3]. *Aspidoras* was described by Ihering [4] as a monotypic genus harboring his new species, *A*. *rochai*. Currently, *Aspidoras* presents 22 valid species which are widespread in many regions of the Brazilian territory. According to Britto [3], *Aspidoras* can be distinguished from *Corydoras* and *Scleromystax* by presenting the following combination of features: (I) posterior portion of mesethmoid wide; (II) frontal fontanel reduced; (III) supraoccipital fontanel present; (IV) opercle compact; (V) ossified portion of pectoral spine strongly reduced, less than half the length of the first branched pectoral-fin ray. Britto [4] also mentioned that *Aspidoras* generally presents smaller eyes than other Corydoradinae and absence of contact between the parieto-supraoccipital and the nuchal plate (except for *A*. *belenos* Britto).

*Aspidoras albater* Nijssen & Isbrücker is described from the upper Tocantins River basin, in Goiás State. Although this species presents a relatively comprehensive description, based on a type-series of more than 10 specimens, and considering that there are several specimens available for examination in museums and collections, its misidentification is very common. Secutti *et al*. [5] provided a brief description of populations of *Aspidoras* from the Anésio-Russão cave system, also drained by the upper rio Tocantins basin, in Goiás State. Additionally, the authors presented a morphological analysis comparing the cave populations with epigean specimens attributed to *A*. *albater* from the same area. Despite the fact that the authors have found some differences between the hypogean and epigean populations, they stated that these differences may be an effect of undergoing a process of differentiation, and since no conspicuous diagnostic feature was observed, they chose the more conservative option, considering these populations as the same species as the epigean population found in the region.

We were able to examine some specimens identified by Secutti *et al*. [5] as *A*. *albater* (MZUSP 40650), corroborating their identification. However, after the examination of the cave specimens analyzed by Secutti *et al*. [5] plus additional specimens also from the Anésio-Russão cave system, and epigean specimens of the region, the holotype and four paratypes of *A*. *albater*, it was possible to refute the identification of the hypogean populations as *A*. *albater*, revealing it as a new species, which is clearly different from all congeners. Here we present the description of this cave-dwelling species of *Aspidoras*, the first troglobitic Callichthyidae catfish, restricted to two caves of a single cave-system from central Brazil.

## Material and methods

Collection permit was granted by the Instituto Chico Mendes de Biodiversidade to MEB #20165). The live specimens were anesthetized in a Benzocaine solution with 50 ppm concentration, following the posteriorly fixed in 10% formalin, and then transferred to 70% ethanol. These steps are approved by the ethical use of animals commission of Universidade Federal de São Carlos (CEUA/UFSCar) which considerers animal welfare laws. Measurements were obtained using digital caliper to the nearest millimeter. Morphometric and meristic data were taken following Reis [6] with modifications of Tencatt *et al*. [7], and are all available at https://figshare.com/articles/A_mephisto_xlsx/4583584. Least interorbital distance, horizontal orbit diameter and snout length were measured only in specimens with distinguishable orbit. Morphometrics are reported as proportion of standard length (SL) or as proportions of head length (HL). Homology of barbels follows Britto & Lima [8]. For the osteological analysis, some specimens were cleared and stained (c&s) according to the protocol of Taylor & Van Dyke [9]. Osteological terminology was based on Reis [10], except by using parieto-supraoccipital instead of supraoccipital [11], compound pterotic instead of pterotic-supracleithrum [12], and scapulocoracoid instead of coracoid [13]. Nomenclature of latero-sensory canals and preopercular pores according to Schaefer & Aquino [14] and Schaefer [15], respectively. The supra-preopercle *sensu* Huysentruyt & Adriaens [16] were treated here as a part of the hyomandibula according to Vera-Alcaraz [17]. Vertebral counts include only free centra, with the compound caudal centrum (preural 1+ ural 1) counted as a single element. In the description, numbers in parenthesis represent the total number of specimens with those counts. Numbers with an asterisk refer to the counts of the holotype.

Comparative data of *Aspidoras brunneus* Nijssen & Isbrücker was obtained through its original description [18] and/or high resolution photographs of the holotype, which are available for examination through the All Catfish Inventory website [19].

**Fig 1:**
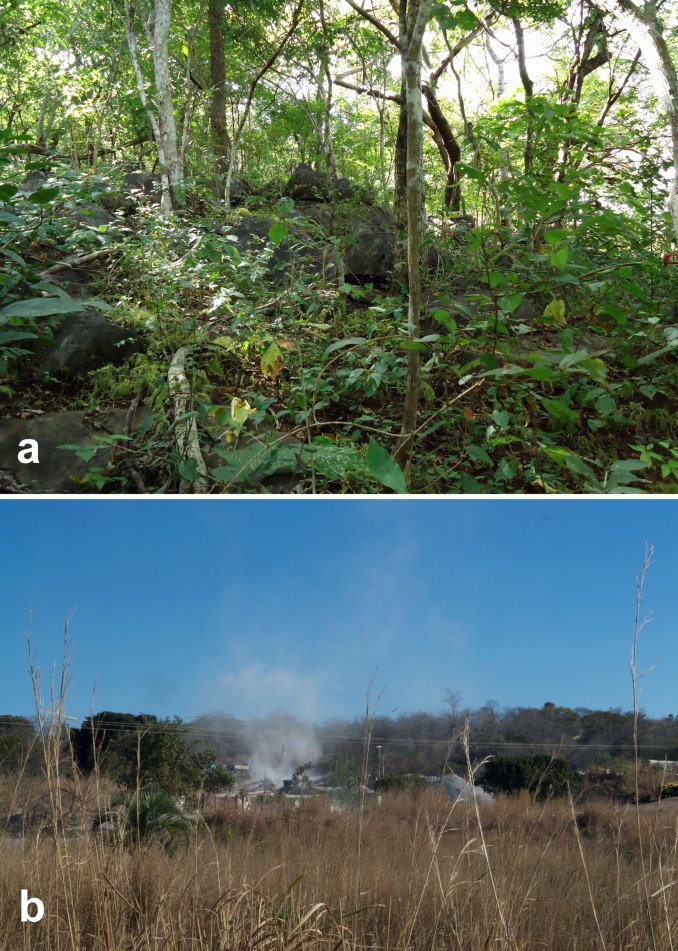
Karst outcrops and threats observed at Posse region, Goiás Brazil. a, Typical karst outcrops observed in the Posse region; b, deforestation in the front and mining in the back, observed close to the Anésio III cave in August of 2016. Photographs: Maria E. Bichuette.

Anésio-Russão cave system is formed by a limestone rock (a karstified rock *sensu* [20]) and is located at Posse municipality, northeastern Goiás state, central Brazil. The karst from northeastern Goiás is a regional expression of the Bambuí geomorphological unit [21] with small outcrops frequently hidden in the vegetation (Fig 1A). The region is inserted on the Cerrado morphoclimatic domain [22], and the climate is tropical semi-humid, with 5–6 dry months [23]. The karst is inserted mostly at the eastern margin of the Paranã river, a tributary of the Upper Tocantins river. There are no surface drainages in the surroundings of the cave-system. Anésio-Russão cave system is parallel to the Posse municipality, far ca. 2–3 km, and is suffering impacts from urban growth such as aquifer pollution (by domestic sewage), deforestation of surroundings and small mining projects (Fig 1B).

**Fig 2:**
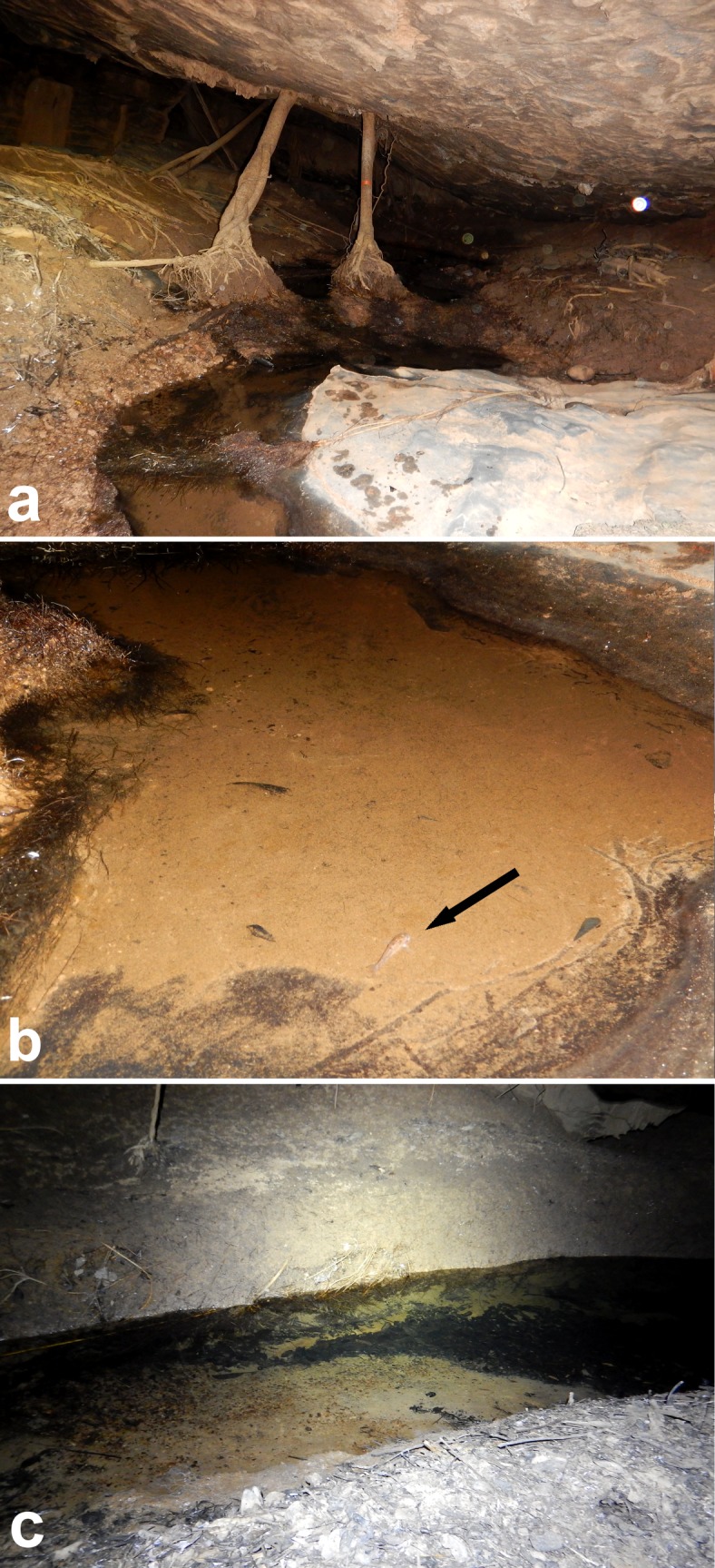
Habitat of *Aspidoras mephisto* at Anésio III cave, Posse Goiás, Brazil, showing (a), a small stretch of the drainage showing the roots reaching the water, lentic and formed by small roots and silt (Photograph: Maria E. Bichuette); (b), a pool with rocky in the border and silty-bottom, the black arrow indicates an adult specimen (Photograph: Maria E. Bichuette); and (c), small stretch stream where the juvenile specimens were observed with a large amount of organic matter in the margin (Photograph: Maria E. Bichuette).

The cave-system has, until now, around 6.000 m of mapped passageways (E. C. Igual, pers. comm.), with subterranean drainages characterized by small to medium size pools and small slow/moderate water current places allied to large amount of organic matter and, for Anésio III cave, several roots crossing the ceiling and reaching the water (Fig 2A and 2B).

Abiotic variables of water (measured on April 2013 –beggining of dry season) show some values typical of kart drainages (pH 7.8, Conductivty 0.514 mS.cm^-1^, Dissolved Oxygen 3.9 mg.l^-1^, Salinity 0.03%, Temperature 22.3°C and Total Dissolved Solutes 0.329 g.l^-1^) and quitely different from epigean drainages (pH 6.9, Conductivty 0.248 mS.cm^-1^, Dissolved Oxygen 6.9 mg.l^-1^, Salinity 0.01%, Temperature 26.7°C and Total Dissolved Solutes 0.162 g.l^-1^).

### Nomenclatural acts

Institutional abbreviations follow Sabaj [24]. The electronic edition of this article conforms to the requirements of the amended International Code of Zoological Nomenclature, and hence the new names contained herein are available under that Code from the electronic edition of this article. This published work and the nomenclatural acts it contains have been registered in ZooBank, the online registration system for the ICZN. The ZooBank LSIDs (Life Science Identifiers) can be resolved and the associated information viewed through any standard web browser by appending the LSID to the prefix “http://zoobank.org/”. The LSID for this publication is: urn:lsid:zoobank.org:pub: A21D7F96-7F10-4E1C-AC7E-2F4551E26C83. The electronic edition of this work was published in a journal with an ISSN, and has been archived and is available from the following digital repositories: PubMed Central, LOCKSS.

## Results

*Aspidoras mephisto*, new species

urn:lsid:zoobank.org:act:10B01ED0-CF81-4E2C-9D9F-EF094CE3509C (Figs 3–9, 10–12,13A and 14)

**Fig 3:**
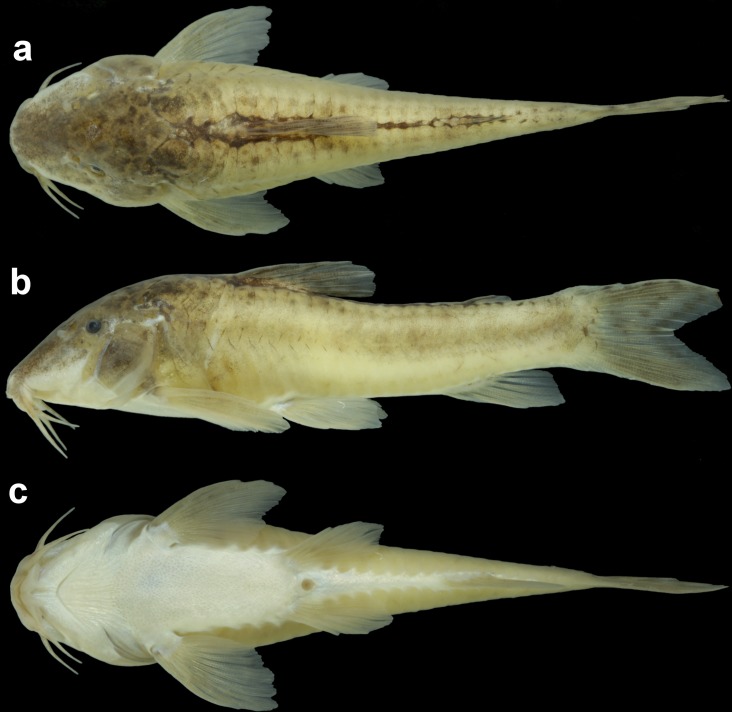
*Aspidoras mephisto*, holotype, MNRJ 48268, 45.6 mm SL, in dorsal (a), lateral (b) and ventral (c) views.

**Fig 4:**
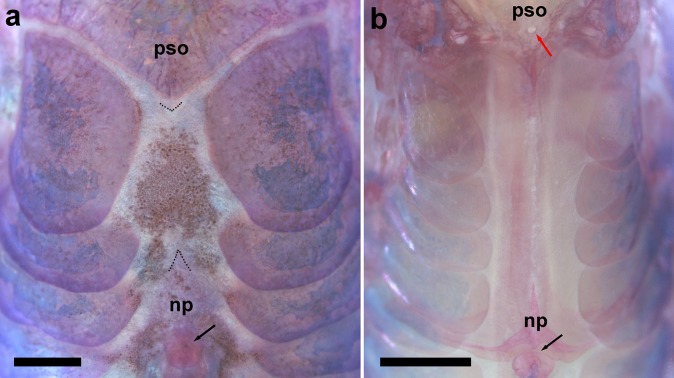
Predorsal portion of trunk of (a) *Aspidoras mephisto*, c&s paratype, NUP 18760, 31.7 mm SL, showing the absence of contact between the process of the parieto-supraoccipital and nuchal plate, which is entirely covered by a thick layer of skin, and (b) *Aspidoras velites* c&s, LIRP 4479, 25.1 mm SL, showing the more posterior parieto-supraoccipital fontanel (red arrow). Dotted lines in (a) indicate the limits of the tip of the posterior process of the parieto-supraoccipital, which is covered by thick layer of skin, and anterior tip of the nuchal plate. Abbreviations: np: nuchal plate, pso: parieto-supraoccipital. Black arrows indicate the dorsal-fin spinelet in (a), and where it should be in (b). Scale bar = 1.0 mm.

**Fig 5:**
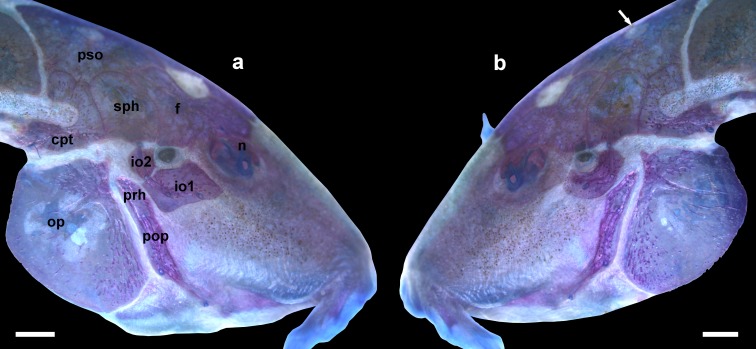
Lateral view the head of *Aspidoras mephisto*, c&s paratype, NUP 18760, 31.7 mm SL, showing right (a) and left (b) sides of its head; io2 absent only on the left side of the head. The white arrow in (b) indicates the parieto-supraoccipital fontanel. Abbreviations: io1: infraorbital 1, io2: infraorbital 2, sph: sphenotic, cpt: compound pterotic, f: frontal, n: nasal, op: opercle, prh: posterodorsal ridge of hyomandibula, pop: preopercle, pso: parieto-supraoccipital. Scale bar = 1.0 mm.

**Fig 6:**
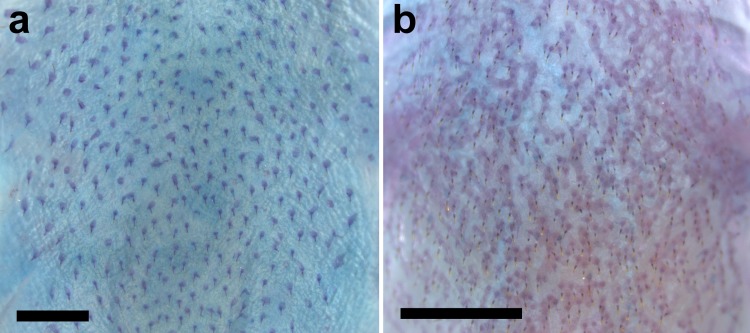
Ventral surface of trunk of (a) *Aspidoras mephisto* c&s paratype, NUP 18760, 31.7 mm SL, showing clearly smaller and rounded or irregular platelets, and (b) *Aspidoras velites* c&s, LIRP 4479, 25.1 mm SL, showing clearly larger and striated platelets. Scale bar = 1.0 mm.

**Fig 7:**
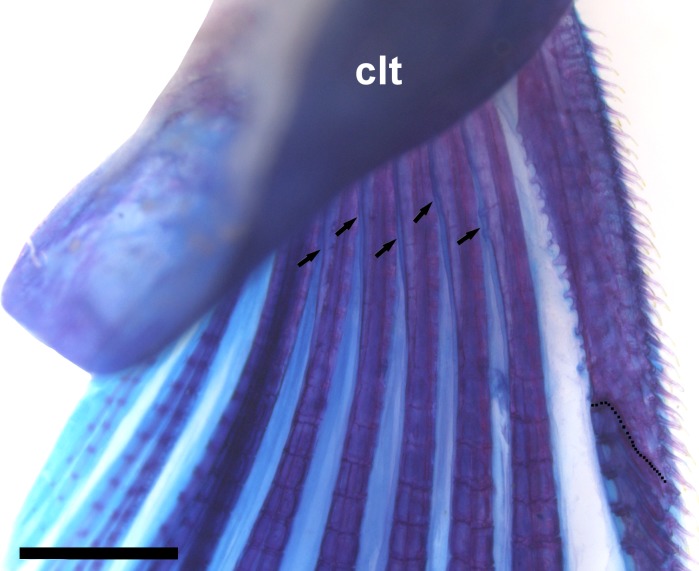
Right pectoral-fin spine of *Aspidoras mephisto*, c&s paratype, NUP 18760, 31.7 mm SL, showing the serration pattern of its posterior margin. Arrows indicate the small laminar expansions on base of first branched rays; dotted line indicates the limit of the conspicuously ossified portion of the pectoral spine. Abbreviations: clt: cleithrum. Scale bar = 1.0 mm.

**Fig 8:**
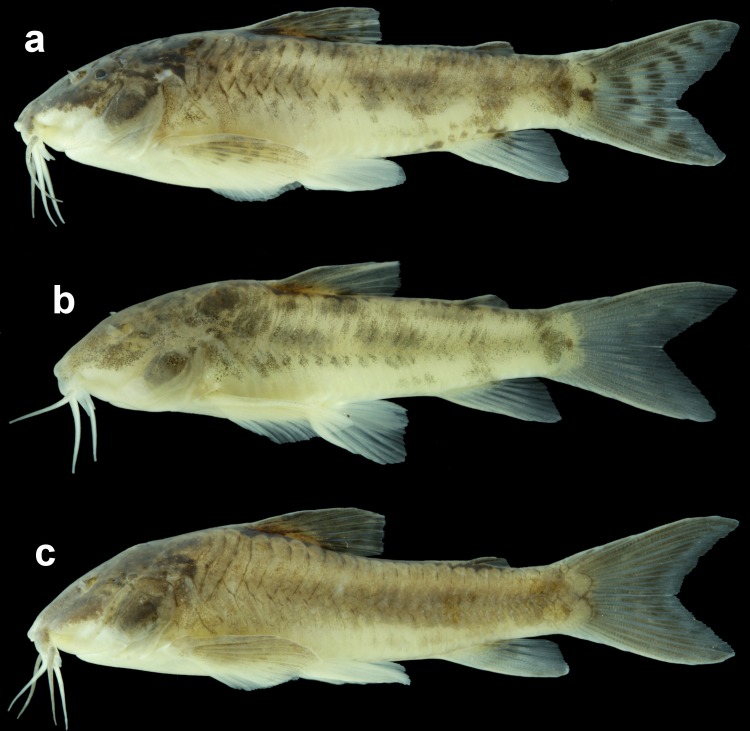
Variations in the general color pattern of *Aspidoras mephisto*, showing the more intensely pigmented pattern, with distinct midlateral blotches (a, NUP 18759, 34.7 mm SL); an intermediary pigmented pattern, with more diffuse but distinct midlateral blotches (b, ZUFMS-PIS 4965, 26.0 mm SL); and another possible intermediary pattern, with a longitudinal diffuse brownish stripe along midline of flank instead of midlateral blotches (c, NUP 18759, 34.9 mm SL).

**Fig 9:**
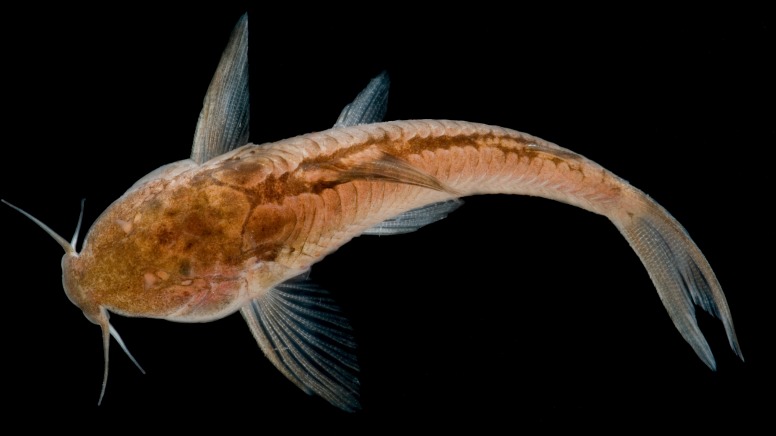
Live uncatalogued specimen of *Aspidoras mephisto* from the Anésio III cave, Posse, Goiás, Brazil. Photograph: Danté Fenolio.

**Fig 10:**
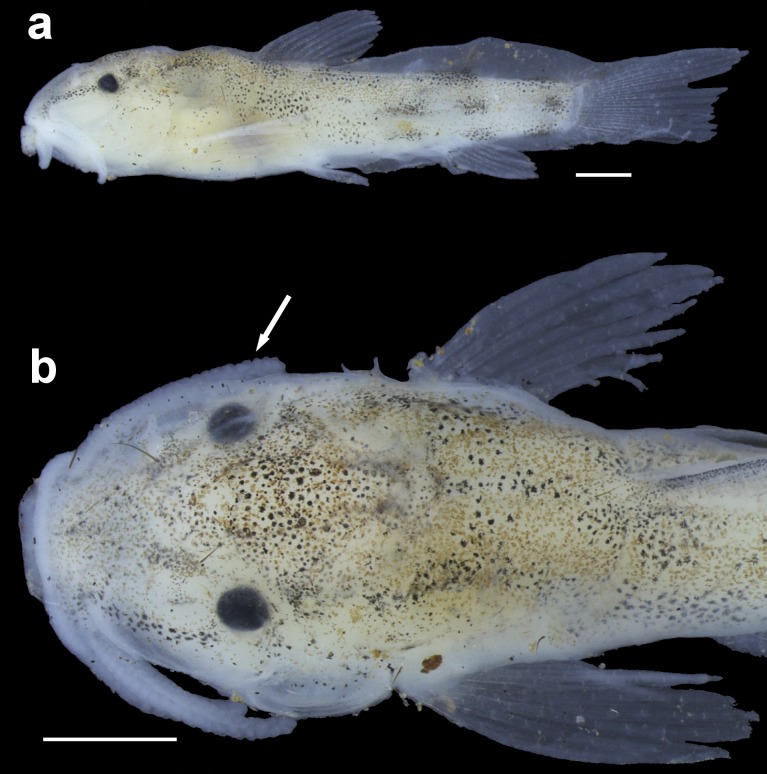
Juvenile paratype of *Aspidoras mephisto*, LESCI 333, 19.0 mm SL, in lateral view (a) and detail of the dorsal view of the anterior portion of the body (b), showing general morphology and color pattern. Arrow indicates the well-developed papillae on barbel. Scale bar = 1.0 mm. Photograph: Luciana B. R. Fernandes.

**Fig 11:**
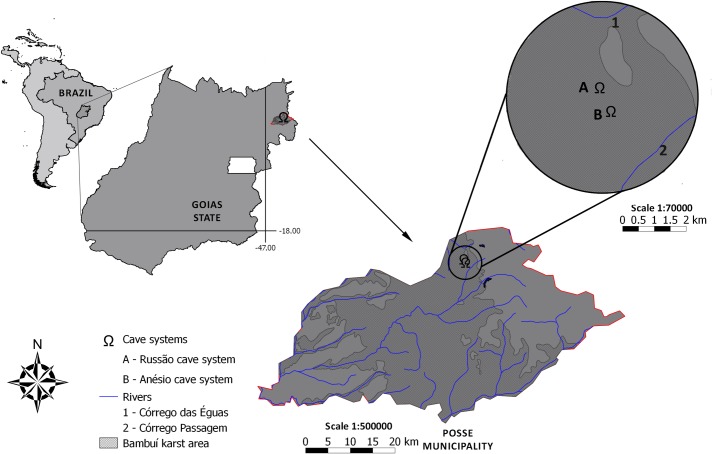
Map of the type-locality of *Aspidoras mephisto*, showing the Anésio-Russão cave-system, Posse, Goiás Brazil. Author: Diego M. von Schimonsky.

**Fig 12:**
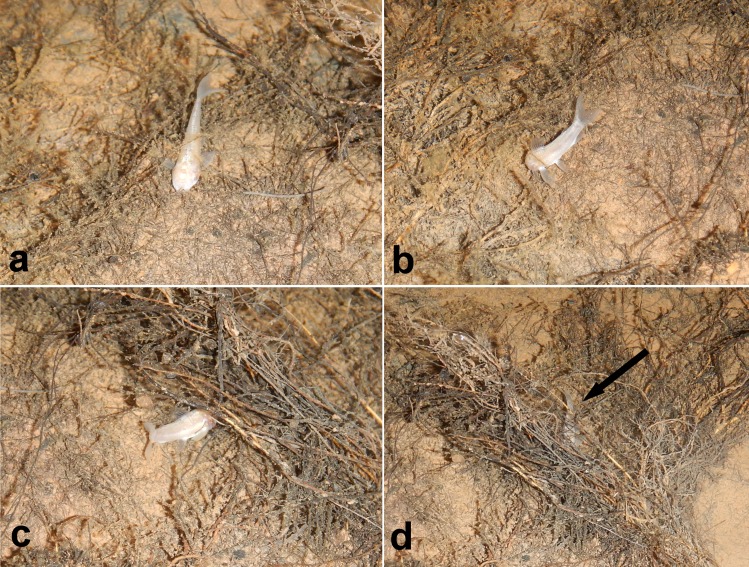
Foraging habitat observed for *Aspidoras mephisto*. (a), touching the bottom parallel to; (b), touching the silty-bottom + roots showing a 60° angle to; (c), starting to foraging under the small roots; and (d), foraging behavior under the roots (arrow). Photographs: Maria E. Bichuette.

**Fig 13:**
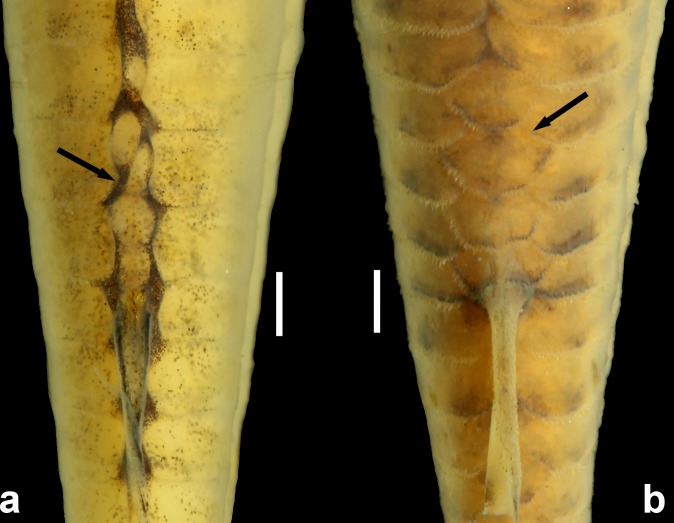
Preadipose azygous plates (arrows) of (a), *Aspidoras mephisto*, holotype, MNRJ 48268, 45.6 mm SL, and (b) *Aspidoras fuscoguttatus*, DZSJRP 14959, 35.1 mm SL, showing the clearly smaller plates in the new species. Scale bar = 1.0 mm. Photographs: Bruno F. dos Santos.

**Fig 14:**
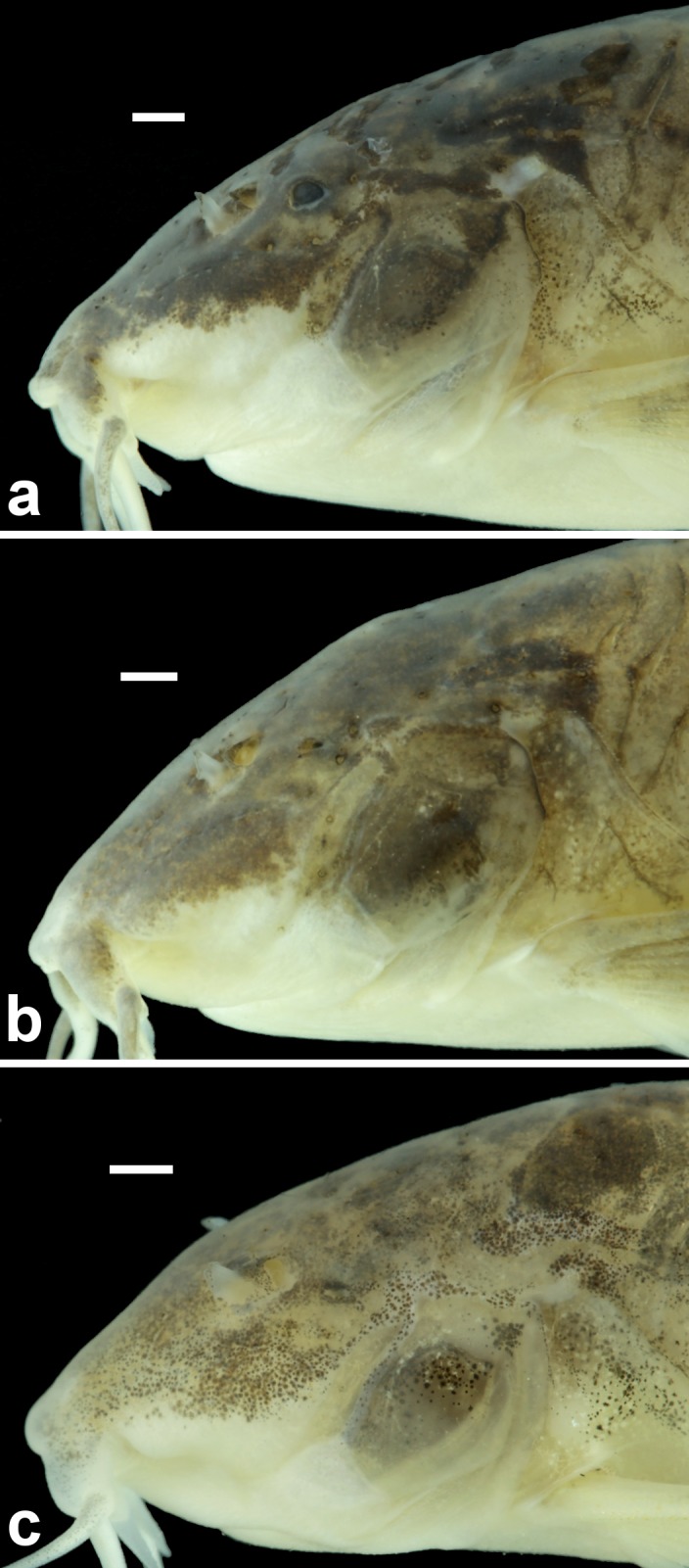
Detail of the head of *Aspidoras mephisto* in lateral view, showing the variations on eye morphology. In (a), NUP 18759, 34.7 mm SL orbit is slightly reduced, clearly smaller than in congeners; in (b), NUP 18759, 34.9 mm SL, orbit is conspicuously reduced but distinguishable and externally opened; and in (c), ZUFMS-PIS 4965, 26.0 mm SL, orbit is strongly reduced, indistinguishable and not externally opened. Scale bar = 1.0 mm.

### Type specimens

Holotype. MNRJ 48268, 45.6 mm SL, Brazil, Goiás, Posse, Anésio III cave, Tocantins River basin, 14°05’27.7”S, 46°22’59.6”W, 7 Apr 2007, E. Trajano & M. E. Bichuette.

Paratypes. All from Brazil, Goiás, Posse, Tocantins River basin. LESCI 246, 7, 18.8–31.2 mm SL; LESCI 304, 2, 33.1–31.3 mm SL; LESCI 306, 1, 33.9 mm SL; NUP 18759, 2, 34.7–34.9 mm SL; NUP 18760, 2 c&s, 29.5–31.7 mm SL, Russão II cave, 14°05’05.3”S, 46°23’07.1”W, 23 Apr 2011, M. E. Bichuette, P. P. Rizzato & J. E. Gallão. LESCI 332, 9, 19.0–27.0 mm SL, Russão II cave, 14°05’05.3”S, 46°23’07.1”W, 23 May 2015, J. E. Gallão. LESCI 333, 1, 19.0 mm SL, Anésio III cave, 14°05’27.7”S, 46°22’59.6”W, 5 Aug 2016, M. E. Bichuette. MCP 40634, 1, 29.0 mm SL, uncertain locality, probably from the Anésio III or the Russão III cave, 7 Apr 2008, E. Trajano. MZUSP 97905, 13, 13.3–37.1 mm SL, 2 c&s, 27.3–30.9 mm SL, Anésio III cave, 14°05’27.7”S, 46°22’59.6”W, 1 Apr 2007, E. Trajano. MZUSP 97906, 8, 19.4–33.8 mm SL, 2 c&s, 25.7–30.3 mm SL, Russão II cave, 14°05’05.3”S, 46°23’07.1”W, 1 Apr 2007, S. Secutti. ZUFMS-PIS 4965, 1, 26.0 mm SL, Russão II cave, 14°05’05.3”S, 46°23’07.1”W, 23 May 2015, J. E. Gallão.

### Diagnosis

*Aspidoras mephisto* can be distinguished from all of its congeners by presenting conspicuous reduction of pigmentation (*vs*. well-developed pigmentation). Another useful feature to recognize the new species is the presence of conspicuously reduced orbit in most specimens (*vs*. clearly larger orbit). Additionally, *Aspidoras mephisto* can be distinguished from its congeners, with exception of *A*. *velites* Britto, by having conspicuously smaller preadipose azygous plates (*vs*. conspicuously larger); it differs from *A*. *velites* by dorsal-fin spinelet present (Fig 4A) (*vs*. absent, Fig 4B); parieto-supraoccipital fontanel located mesially on bone (Fig 5) (*vs*. located posteriorly on bone, close to origin of posterior process, Fig 4B); absence of conspicuous dark brown or black stripe from anteroventral portion of eye to upper lip lateral area (*vs*. presence); and absence of striated small platelets on ventral surface of trunk (Fig 6A) (*vs*. presence, Fig 6B). The new species can also be distinguished from its congeners, with exception of *A*. *albater* Nijssen & Isbrücker, *A*. *belenos* Britto, *A*. *carvalhoi* Nijssen & Isbrücker, *A*. *eurycephalus* Nijssen & Isbrücker, *A*. *fuscoguttatus* Nijssen & Isbrücker, *A*. *lakoi* Miranda Ribeiro, *A*. *maculosus* Nijssen & Isbrücker, *A*. *marianae* Leão, Britto & Wosiacki, *A*. *menezesi* Nijssen & Isbrücker, *A*. *poecilus* Nijssen & Isbrücker, *A*. *raimundi* (Steindachner), *A*. *rochai* Ihering, *A*. *spilotus* Nijssen & Isbrücker and *A*. *virgulatus* Nijssen & Isbrücker, by the presence of infraorbital 1 generally with well-developed ventral laminar; or moderately-developed (*vs*. poorly developed). The new species can be distinguished from *A*. *belenos*, *A*. *fuscoguttatus*, *A*. *lakoi*, *A*. *maculosus*, *A*. *marianae*, *A*. *menezesi*, *A*. *poecilus*, *A*. *raimundi*, *A*. *spilotus* and *A*. *virgulatus* by presenting poorly-developed serrations on posterior margin of pectoral spine (*vs*. strongly well-developed serrations in *A*. *virgulatus*; from moderately- to well-developed serrations in remaining species); from *A*. *albater* and *A*. *eurycephalus* by presenting dorsal fin, even in conspicuously colored specimens, with only dark brown or black chromatophores concentrated on rays, forming spots in some specimens; membranes hyaline; or presence of isolated dark brown or black chromatophores on membranes, not forming any conspicuous pattern (*vs*. dorsal fin with spots; conspicuous concentration of dark brown or black chromatophores on some areas of membranes, forming larger dark brown or black patches), and by having inner laminar expansion of infraorbital 1 moderately developed (*vs*. well-developed or extremely well-developed); and from *A*. *carvalhoi* and *A*. *rochai* by having nuchal plate not externally visible (*vs*. externally visible).

### Description

Morphometric data presented in Table 1. Head laterally compressed with convex dorsal profile; somewhat trapezoidal in dorsal view. Snout relatively well-developed and pointed or moderately developed and more rounded. Head profile slightly convex to convex from tip of snout to anterior nares; ascending slightly convex from this point to dorsal-fin origin. Profile slightly convex along dorsal-fin base. Postdorsal-fin body profile slightly concave to adipose-fin spine; slightly concave from this point to caudal-fin base. Ventral profile of body slightly convex from isthmus to pelvic-fin origin; nearly straight from this point to anal-fin origin; slightly concave until caudal-fin base. Body roughly elliptical in cross section at pectoral girdle, gradually becoming more compressed toward caudal fin.

**Table 1:** Morphometric data of holotype and 19 paratypes of *Aspidoras mephisto*. Length of maxillary barbel value is absent in a single paratype. N = number of measured specimens and SD = standard deviation.

	N	Holotype	Low-High	Mean±SD
Standard length (mm)	20	45.6	18.8–45.6	30.4±5.8
	**Percents of standard length**			
Depth of body	20	24.1	23.3–28.2	26.4±1.6
Predorsal distance	20	45.2	44.2–48.4	46.0±1.0
Prepelvic distance	20	47.6	47.0–53.1	50.2±1.6
Preanal distance	20	77.9	77.0–81.6	79.0±1.2
Preadipose distance	20	84.4	81.9–88.5	85.6±1.7
Length of dorsal spine	20	6.1	6.1–13.8	9.7±1.7
Length of pectoral spine	20	10.1	10.0–15.4	12.4±1.4
Length of adipose-fin spine	20	5.0	5.0–9.1	7.6±1.1
Depth of caudal peduncle	20	12.3	11.8–16.0	13.8±1.0
Length of dorsal-fin base	20	14.0	11.7–15.9	13.2±1.0
Dorsal to adipose distance	20	28.7	25.4–29.7	27.7±1.4
Maximum cleithral width	20	25.4	24.0–27.6	25.9±0.9
Head length	20	35.7	33.1–38.3	35.4±1.2
Length of maxillary barbel	19	13.2	12.8–24.8	18.0±2.9
	**Percents of head length**			
Head depth	20	65.6	63.6–74.8	68.8±3.2
Least interorbital distance	8	33.7	33.7–41.3	38.3±2.4
Horizontal orbit diameter	8	9.8	5.9–12.1	9.0±2.1
Snout length	8	53.4	50.5–55.4	52.8±1.8
Least internarial distance	20	20.2	17.1–25.3	21.5±2.3

Eye remarkably variable; orbit, when distinguishable, located dorso-laterally on head and delimited dorsally by lateral ethmoid, frontal and sphenotic; lateral ethmoid not delimiting orbit in some specimens; ventrally by both infraorbitals or only by infraorbital 1. Orbit variable in shape, with rounded, elliptical or irregular aspect; or indistinguishable; crystalline lens present or apparently absent. Anterior and posterior nares close to each other, only separated by flap of skin. Anterior naris tubular. Posterior naris separated from anterodorsal margin of orbit, when distinguishable, by distances ranging from close (distance equal to naris diameter) to relatively distant (distance larger than naris diameter). Mouth small, subterminal, width at least around two and a half times larger than bony orbit diameter, when distinguishable. Maxillary barbel generally moderate in size, not reaching anteroventral limit of gill opening; or relatively long, reaching to anteroventral limit of gill opening. Outer mental barbel slightly larger than maxillary barbel. Inner mental barbel fleshy, with base close to its counterpart. Lower lip moderately developed, forming small semicircular fleshy flap; or more developed, forming triangular expansion. Small rounded papillae covering entire surface of all barbels, upper and lower lips, snout and isthmus.

Mesethmoid short; anterior tip long, larger than 50% of entire bone length (see Britto [4], page 123, character 1, state 0; Fig 1A); posterior portion wide, entirely covered by thick layer of skin. Nasal slender, curved laterally, inner margin with moderately-developed laminar expansion; outer margin with reduced laminar expansion; mesial border generally contacting only frontal; or contacting frontal and mesethmoid. Frontal elongated, relatively thick, with width slightly larger than half of entire length; anterior projection ranging from short to long, with size varying from smaller than, equal to or slightly larger than nasal length. Frontal fontanel relatively small, roughly rhomboid; posterior tip extension not entering anterior margin of parieto-supraoccipital. Parieto-supraoccipital wide, posterior process conspicuously short, not contacting nuchal plate; posterior tip covered by thick layer of skin. Parieto-supraoccipital fontanel small, roundish or horizontally elliptical; located mesially on parieto-supraoccipital.

Two laminar infraorbitals with minute odontodes; infraorbital 1 large, ventral laminar expansion generally well developed; or moderately developed; anterior portion with poorly- or moderately-developed expansion (Fig 5); inner laminar expansion moderately developed; infraorbital 2, when present, small, generally compact; or slightly longer; with posterior laminar expansion moderately developed; posteroventral margin not directly contacting posterodorsal ridge of hyomandibula, dorsal tip contacting only sphenotic (Fig 5); inner laminar expansion moderately developed. Posterodorsal ridge of hyomandibula close to its articulation with opercle oblong; exposed, relatively slender; dorsal ridge of hyomandibula between compound pterotic and opercle covered by thick layer of skin; exposed areas bearing small odontodes. Interopercle entirely or almost entirely covered by thick layer of skin, somewhat triangular, anterior projection moderately- or well-developed. Preopercle relatively slender, elongated, minute odontodes sparse on external surface. Opercle roughly compact in shape, width conspicuously larger than half of its length; free margin convex; posterodorsal region with smoothly concave area in some specimens; without serrations and covered by small odontodes.

Four branchiostegal rays decreasing in size posteriorly. Hypobranchial 2 somewhat triangular, tip ossified and directed towards anterior portion, posterior margin cartilaginous; ossified portion well developed, about twice size of cartilaginous portion; or conspicuously well developed, about triple size of cartilaginous portion. Five ceratobranchials with expansions increasing posteriorly; ceratobranchial 1 with small process on anterior margin of mesial portion; ceratobranchial 3 with continuous postero-lateral margin; ceratobranchial 5 toothed on postero-dorsal surface, 25 to 33 (6) teeth aligned in one row. Four epibranchials with similar size; epibranchial 2 slightly larger than others, with small pointed process on laminar expansion of posterior margin; epibranchial 3 with somewhat triangular uncinate process on laminar expansion of posterior margin. Two wide pharyngobranchials (3 and 4), pharyngobranchial 3 with triangular laminar expansion on posterior margin. Upper tooth plate oval; 29 to 42 (6) teeth generally aligned in two rows on postero-ventral surface; teeth aligned in three rows in specimen LESCI 246, 31.7 mm SL.

Lateral-line canal entering neurocranium through compound pterotic, branching twice before entering sphenotic: pterotic branch with a single pore; preoperculomandibular branch conspicuously reduced, with a single pore opening close to postotic main canal. Sensory canal continuing through compound pterotic, entering sphenotic as temporal canal, which splits into two branches: one branch giving rise to infraorbital canal, other branch entering frontal through supraorbital canal, both with single pore. Supraorbital canal branched, running through nasal bone. Epiphyseal branch of supraorbital canal relatively long; pore opening close to frontal fontanel. Nasal canal with three openings, first on posterior edge, second on posterolateral portion and third on anterior edge; second pore fused with first pore in some specimens. Infraorbital canal running through entire second infraorbital (when present), extending to infraorbital 1 and opening into one, two or four pores. Preoperculomandibular branch giving rise to preoperculo-mandibular canal, which runs through entire preopercle with three openings, leading to pores 3, 4, and 5, respectively; pore 3 opening at posterodorsal ridge of hyomandibula in some specimens.

Dorsal fin triangular, located just posterior to third or fourth dorsolateral body plate. Dorsal-fin rays II,7 (2), II,8* (18), posterior margin of dorsal-fin spine smooth. Nuchal plate poorly developed; entirely covered by thick layer of skin; spinelet conspicuously short (Fig 4A); entirely covered by thick layer of skin or partially exposed; spine poorly developed, adpressed distal tip not reaching to origin of last dorsal-fin branched ray; anterior margin with small odontodes. Pectoral fin triangular, its origin just posterior to gill opening. Pectoral-fin rays I,9; last pectoral-fin soft ray simple in only one side of body in specimen LESCI 246, 31.7 mm SL; posterior margin of pectoral spine with 9 to 14 poorly-developed serrations along almost its entire length; small region just posterior to origin of spine lacking serrations; serrations generally slightly directed towards tip of the spine; some serrations perpendicularly directed or slightly directed towards origin of spine; presence of bifid serrations in some specimens; base of first branched rays with small laminar expansions on its inner margin; laminar expansions with irregular margins, forming pointed structures, in some specimens (Fig 7); anteroventral portion of cleithrum partially exposed; posterolateral portion of scapulocoracoid reduced, covered by relatively thick layer of skin; externally visible; minute odontodes sparse on exposed areas. Pelvic fin oblong, located just below fourth ventrolateral body plate, and at vertical through third or fourth branched dorsal-fin ray. Pelvic-fin rays i,5. Adipose fin somewhat triangular, separated from base of last dorsal-fin ray by nine or 10 dorsolateral body plates. Anal fin somewhat triangular, located just posterior to 13^th^ or 14^th^ ventrolateral body plates, and at vertical through region of preadipose platelets. Anal-fin rays ii,5,i* (6), ii,6 (14). Caudal-fin rays i,11,i (1), i,12,i* (19), generally six dorsal and ventral procurrent rays; bilobed, dorsal lobe generally slightly larger than ventral lobe; ventral lobe slightly larger than dorsal lobe in holotype.

Generally, two laterosensory canals on trunk; first ossicle tubular, second ossicle laminar; only one canal on trunk, laminar ossicle lacking canal in some specimens. Body plates with minute odontodes scattered over exposed area, a conspicuous line of odontodes confined on posterior margins; dorsolateral body plates 26* (13), 27 (7); ventrolateral body plates 23* (9), 24 (11); dorsolateral body plates along dorsal-fin base 5 (4), 6* (16); dorsolateral body plates between adipose- and caudal-fin 7 (1), 8* (14), 9 (5); preadipose platelets 3 (2), 4 (3), 5* (4), 6 (6), 7 (3), 8 (1), 11 (1); small platelets covering base of caudal-fin rays; small platelets disposed dorsally and ventrally between junctions of lateral plates on posterior portion of caudal peduncle. Ventral surface of trunk generally densely covered by small platelets (Fig 6A); or less densely disposed platelets.

Vertebral count 25 (6); ribs 6 (2), 7 (4) first pair conspicuously large; complex vertebra slender in shape.

#### Color in alcohol

All specimens with conspicuously reduced pigmentation compared to all congeners. Color pattern remarkably variable regarding flank midline pattern and intensity of pigmentation (Figs 3 and 8); ground color of body brownish yellow in specimens preserved for a longer time; whitish yellow just after preservation. Dorsal and lateral portion of head covered by black or dark brown chromatophores; conspicuously concentrated chromatophores in some specimens. Naked area on dorsal region of body, between counterparts of dorsolateral body plates, with concentration of black or dark brown chromatophores. Dorsolateral body plates covered by black or dark brown chromatophores, generally more concentrated on first plate. Dorsal half of ventrolateral body plates covered by black or dark brown chromatophores; ventral half of plates generally with chromatophores only posteriorly to pelvic-fin origin. Post-anal naked area on ventral portion of body, between counterparts of ventrolateral body plates, with concentration of black or dark brown chromatophores in some specimens. Posterior margin of dorso- and ventrolateral body plates with conspicuous concentration black or dark brown chromatophores, generally more evident along flank midline and on anterior portion of body, in some specimens. Flank midline with longitudinal series of four or five diffuse black or dark brown and somewhat rounded or irregular blotches, first below dorsal fin, second and third, when present, between dorsal and adipose fins, fourth below adipose fin, and fifth on posterior portion caudal peduncle; blotches fused, forming diffuse longitudinal stripe in some specimens. Dorsal-fin rays brownish; presence of dark brown or black blotches on rays in some specimens; membranes hyaline; presence of isolated dark brown or black chromatophores in some specimens. Dorsal portion of pectoral-fin rays brownish; with dark brown or black blotches on rays in some specimens; coloration of pectoral fin generally restricted to the first branched rays; hyaline in some specimens. Pelvic fin hyaline. Anal fin generally almost entirely hyaline, with concentration of black or dark brown chromatophores on region of bases of last two branched rays; with diffuse transversal black or dark brown bar in some specimens. Caudal-fin rays covered by black or dark brown chromatophores; distal margin of caudal fin generally lacking chromatophores; presence of two to five transversal black or dark brown bars in some specimens. Middle portion of caudal-fin base with diffuse or conspicuous small rounded black or dark brown blotch.

#### Color in life

Similar to preserved specimens, but with ground color of body whitish yellow to whitish pink, with head and lateral body showing translucent regions. Chromatophores of blotches and markings on body brown or grey. Eyes generally black. Body covered by greenish yellow iridescent coloration (Fig 9).

#### Juvenile general morphological and color patterns

Based on one paratype of 19.0 mm SL (LESCI 333) in postflexion stage (Fig 10). Body elongate, decreasing in depth posteriorly. Barbels with well-developed papillae; tip not reaching anteroventral limit of gill opening. Dorso- and ventrolateral plates absent. All fins, with exception of adipose, completely formed. Skin membrane from base of last dorsal-fin branched ray to region of caudal-fin procurrent rays; adipose-fin spine encased within membrane. Dorsal- and pectoral-fin spines formed but not conspicuously ossified. All rays segmented. Ground color of body yellowish white. Dorsal portion of body and lateral portion of trunk covered by yellowish brown and black chromatophores. Presence of diffuse stripe formed by conspicuous concentration of black chromatophores from anteroventral margin of orbit to lateral portion of upper lip. Flank midline with longitudinal series of diffuse roundish blotches formed by conspicuous concentration of black chromatophores; first blotch below dorsal fin, second between dorsal fin and adipose-fin spine, third on region of adipose-fin spine, and fourth on base of caudal fin. Conspicuous concentration of black chromatophores on base of adipose spine, dorsal portion of caudal-fin base, and on ventral portion of flank below second, third and fourth midlateral blotches. All fins hyaline.

#### Sexual dimorphism

As expected for the Corydoradinae [4, 25], males of *Aspidoras mephisto* present lanceolate genital papillae (*vs*. narrow).

#### Geographical distribution

The new species is known only from the Anésio-Russão cave system (Anésio III and Russão II caves), part of the upper rio Tocantins basin (Fig 11).

#### Etymology

The epithet “*mephisto*” refers to the shortened name of Mephistopheles, demon from the German folklore. Mephistopheles comes from the Greek by the combination of three words: με (me), a negation, φῶς (phōs), meaning light, and φιλις (philis), meaning loving, literally “not-light-loving”, or the one who does not love the light, making allusion to the subterranean behavior of the new species. A noun in apposition.

#### Habitat, ecological and behavioral notes

*Aspidoras mephisto* shows preference to slow waters (small values of dissolved oxygen), small depths (ca. 0.05 m) and bottom formed by silt, clay and boulders, showing higher values of Total Dissolved Solutes compared to the epigean drainage. Its abundance is relatively high compared to other troglobitic fishes (ca. 50 individuals in pools), and population densities of 5–6 inds.m^-2^. Juvenile individuals were observed along small stretches of the drainage, always in lentic and shallow places, isolated from the adults and frequently under roots (Fig 2C).

*Aspidoras mephisto* forages calmly close to small submersed roots and silty-bottom (Fig 12A and 12B). They use the anterior extremity of the snout exploring the substrate in a parallel position or forming an angle of ca. 60° in relation to the bottom (Fig 12B), sometimes under the small roots (Fig 12C and 12D). The fish showed this behavior throughout the entire observation time (ca. 20 min) and did not show escape and/or avoidance behavior due to lamp-light or other external disturbances (such as the presence of the observer).

Additional observations are showed in a video file (https://www.youtube.com/watch?v=ksrV1af0NZY and https://figshare.com/articles/Aspidoras_mephisto_new_species/4583974), which brings a detailed courtship behavior of *A*. *mephisto* at Anésio III cave. In a first time two individuals are observed close to each other; the smaller one (that we considered the first male) starts to go around the larger (which is supposed the female), and gives gentle touches on the flanks, head and tail. Immediately a third individual (the second male) arrives, and then the three stop parallel in relation to each other. After few seconds, they swim in opposite direction and the female stops again for few seconds. In this occasion, the female swims in direction of the first male, showing a preference for this individual. However, the second male arrives and then the first one swims in an opposite direction, avoiding the contact. At this time there is an approach of the second male, touching abruptly and quickly the female in the flanks and tail, but the female escapes immediately, avoiding the copula. These observations are similar to those observed for Corydoradinae fish (R. McLure, pers. comm.). Several juvenile individuals of *A*. *mephisto* were observed along the drainage, suggesting that the fish were in reproductive period (dry season). This is in contrast to the stated behavior of many troglobitic organisms, that show a reproductive peak in the beginning or middle of the rainy season, when food availability and input is higher. One possible explanation is that the Anésio-Russão cave-system can sustain the population due the large amount of submerged roots, which represent shelter and food source for this catfish (see Fig 12) and, possibly, the food is not the main limiting factor. Captive observations suggest that *A*. *mephisto* has a high life expectancy, a typical characteristic of troglobitic organisms [26]. The holotype was captured in adult stage in April 7, 2007 and was kept alive until its preservation, in April 20, 2016 (nine years in captivity). Additionally, one of the paratypes, MCP 40634, was also captured in adult stage on April 7, 2008 and maintained alive in aquarium until February 13, 2015 (seven years in captivity). Considering that we have no evidence or study showing how much time it takes until they reach the adult stage, we can hypothesize a minimal life expectancy of at least ten years for *A*. *mephisto*.

## Discussion

In a first look, the troglomorphic features can distinguish *A*. *mephisto* from all of its congeners, however, a non-troglomorphic feature is very useful to recognize the new species, the presence of conspicuously smaller preadipose platelets (Fig 13), that is shared only with *A*. *velites*, from which it can be clearly distinguished (see Diagnosis). Despite Secutti *et al*. [5] have considered *A*. *mephisto* as hypogean populations of *A*. *albater*, we discarded this possibility based in conspicuous differences between both species, such as the peculiar pattern of the preadipose platelets and the dorsal-fin color pattern and the size of the inner laminar expansion of infraorbital 1 (see diagnosis). Even with some degree of overlapping, the infraorbital bones are also useful to distinguish both species, since most *A*. *mephisto* presents well developed ventral laminar expansion of infraorbital 1 and relatively thick and compact infraorbital 2, when present, contrary to *A*. *albater*, which generally present ventral laminar expansion of infraorbital 1 ranging from poorly- to moderately developed, and infraorbital 2 slender and elongated. Few specimens of *A*. *mephisto* present moderately developed ventral laminar expansion of infraorbital 1 and more elongated infraorbital 2 (when present) and, in these cases, the features mentioned in the diagnosis can clearly differ both species.

Subterranean organisms categorized as troglobites are incapable to form epigean source populations and thus are restricted to subterranean habitats [27]. These organisms are specialized to the subterranean life and some character states related to this selective regime (called troglomorphisms) are sometimes expressed, mainly the reduction of eyes and melanic pigmentation [28] and are highly homoplastic. It was possible to recognize at least four orbital stages of development: (I) higher development of orbit, similar to congeners (Fig 3); (II) orbit slightly reduced, clearly smaller than in congeners (Fig 14A); (III) orbit conspicuously reduced but distinguishable and externally opened (Fig 14B); and (IV) orbit strongly reduced, indistinguishable and not externally opened (Fig 14C). From the 51 examined specimens, 14 presented stage I (≈ 27.5%), 10 with stage II (≈ 19.6%), 12 with stage III (≈ 23.5%), and 15 in stage IV (≈ 29.4%), showing that more than 70% of the population present orbit ranging from clearly smaller to strongly reduced.

Regarding intensity if pigmentation, all specimens presented clear reduction of pigmentation, observed in three stages: (I) reduced but slightly more pigmented than most conspecific specimens (Fig 3A); (II) slightly less pigmented than stage I (Fig 3B and 3C); and (III) strongly less pigmented than stages I and II (Figs 9 and 12). We were able to check these stages in 44 specimens, of which only one specimen presented stage I (≈ 2.3%), 31 presented stage II (≈ 70.0%), and 12 presented stage III (≈ 27.7%). This mosaic of characters (eyes and melanic pigmentation variability) are frequently observed in the eight troglobitic fishes from northeastern Goiás [29, 30, 31, 32, 33, 34] and also for other troglobitic catfishes from Brazil (see [35]), and, as observed for *A*. *mephisto*, the reduced orbits and the reduced pigmentation are more frequent than regular eyes and pigmented individuals. These observations can be considered as an unmarked specialization related to the cave life (low degree of troglomorphism) and, *sensu* some authors [36], can be related to a short time of isolation in subterranean environments.Despite the fish collections effort in the epigean drainages close to Posse and in São Domingos karst area, no specimen of *A*. *mephisto* was recorded [29]. The region presents the highest subterranean ichthyofauna from South America, with nine described species (eigth belongs to the Siluriformes order: *Ituglanis*, *Pimelodella* and *Ancistrus* genera; and one to Gymnotiformes order: *Eigenmannia* genus) [29] and more two under description (Siluriformes: *Cetopsorhamdia* and *Ituglanis* genera), with a historical of collections since 1980's years, which implies an expressive sampling effort, in the subterranean environment and surface drainages (M. E. Bichuette, pers. obs). Therefore, the absence of epigean drainages close to Anésio-Russão cave system and populations of *A*. *mephisto* in the closest epigean drainages, along with the observed troglomorphisms (eyes conspicuously reduced in most specimens and reduced pigmentation the body in all specimens, including specimens kept in aquarium), categorize *A*. *mephisto* as a troglobitic species, with only two subpopulations.

Considering the International Union for Conservation of Nature criteria for fauna threatened category, we proposed that *A*. *mephisto* must be considered at least as EN (Endangered), which the assumptions are: B2abiii (occupation area lower 500 km^2,^ no more than 5 localities and habitat quality showing continuous decrease) [37]. *Aspidoras mephisto* has an occupation area of approximately 6 km^2^, the surroundings of cave-system (two localities) is deforested for pastures and urban growth, the drainages are suffering discharge of domestic sewage, and the Anésio-Russão cave system currently has no legal protection by Brazilian environmental laws. Therefore, the combination of these facts could lead the new species to a worst threatened status in the near future, and it must be strongly considered to propose conservation actions for the species itself (e.g. long term population studies), for the cave system as whole and for the vegetation in the caves surroundings.

## Comparative material

All from Brazil. *Aspidoras albater*: LBP 15302, 2, 28.9‒33.9 mm SL, Goiás, da Lapa River; MZUSP 40650, 9 of 53, 16.6‒27.3 mm SL, Goiás, stream tributary to the Manso River. MZUSP 55967, 9, 18.7‒28.0 mm SL, Goiás, da Lapa River sinkhole. MZUSP 12991, holotype, 34.2 mm SL, Goiás, Tocantinzinha River near São João da Aliança; MZUSP 12992, 4, paratypes, 25.2–31.0 mm SL, Goiás, Tocantinzinha River near São João da Aliança. *Aspidoras belenos*: MNRJ 12433, holotype, 27.8 mm SL, Mato Grosso, creek at Primavera do Leste—Paranatinga road, 82 km N from Primavera do Leste; MZUSP 51208, 3, paratypes, 15.0‒26.6 mm SL, Mato Grosso, creek at Primavera do Leste—Paranatinga road, 82 km N from Primavera do Leste. *Aspidoras carvalhoi*: MNRJ 5230, holotype, 25.4 mm SL, Ceará, Canabrava Reservoir. *Aspidoras depinnai*: MZUSP 56214, holotype, 32.5 mm SL, Pernambuco, creek at Amaraji-Primavera road. UFPB 6194, 6, 21.8–35.5 mm SL, 1 c&s, 28.0 mm SL, Pernambuco, do Meio Creek. *Aspidoras eurycephalus*: MNRJ 13080, 8, 10.8–34.5 mm SL, 1 c&s, 32.2 mm SL, Goiás, São Bento Creek. *Aspidoras fuscoguttatus*: MZUSP 8573, holotype, 29.5 mm SL, Mato Grosso do Sul, Corguinho Creek, road of Três Lagoas. NUP 11397, 2 c&s, 30.6–30.7 mm SL, Goiás, dos Bois River. *Aspidoras gabrieli*: MPEG 17394, 5 of 139, paratypes, 16.2–26.3 mm SL, Pará, unnamed tributary to the left bank of rio Parauapebas; MZUSP 87674, 12, 12.7–32.9 mm SL, 1 c&s, 31.4 mm SL, Pará, Jacaré Creek. *Aspidoras lakoi*: MNRJ 5292, holotype, 33.4 mm SL, Minas Gerais, small creek at Floresta do Grotão, da Cachoeira Farm. *Aspidoras maculosus*: UFBA 3291, 3 of 5, 25.7–28.6 mm SL, Bahia, Paiaiá River, on the road BA 131, between Saúde and Pindobaçu. *Aspidoras marianae*: CPUFMT 2060, 5, 12.1–27.7 mm SL, 1 c&s, 27.6 mm SL, Pará, unnamed creek. *Aspidoras menezesi*: MCP 47303, 9 of 16, 23.2–28.8 mm SL, 1 c&s of 16, 28.4 mm SL, Ceará, Batateiras River. *Aspidoras microgalaeus*: MZUSP 51209, 1, holotype, 25.7 mm SL, Mato Grosso, small tributary of Culuene River, km 86 of Paranatinga–Canarana road; MZUSP 51210, 4, 19.2–23.6 mm SL, Mato Grosso, small tributary of Culuene River, km 86 of Paranatinga–Canarana road. *Aspidoras pauciradiatus*: INPA 34595, 5 of 22, 18.9–21.9 mm SL, Roraima, uncertain locality, close to Dona Cota community. *Aspidoras poecilus*: UNT 6249, 29 of 46, 20.0–32.8 mm SL, 1 c&s of 46, 29.3 mm SL, Tocantins, Manduca Stream. *Aspidoras psammatides*: MNRJ 28407, holotype, 25.7 mm SL, Bahia, Caldeirão River; UNT 9604, 18 of 40, 16.4–24.2 mm SL, 2 c&s of 40, 22.9–26.2 mm SL, Bahia, Roncador River. *Aspidoras raimundi*: UFPB 9418, 19 of 51, 16.5–24.2 mm SL, 2 c&s, 21.0–23.0 mm SL, Piauí, creek under the bridge on the road between São Miguel da Baixa Grande–São Félix do Piauí. *Aspidoras rochai*: MZUSP 2195, lectotype, 38.7 mm SL, Ceará, Fortaleza; MZUSP 2195, 1, paralectotype, 35.5 mm SL, Ceará, Fortaleza. *Aspidoras spilotus*: UFPB 9247, 3 of 7, 35.3–40.3 mm SL, 2 c&s of 7, 27.4–30.5 mm SL, Ceará, das Minas River. *Aspidoras taurus*: MZUSP 57154, holotype, 52.1 mm SL, Mato Grosso, Itiquira River; LBP 1427, 29 of 31, 17.3–40.3 mm SL, 2 c&s of 31, 25.3–36.1 mm SL, Mato Grosso, rio Itiquira. *Aspidoras velites*: MZUSP 74447, holotype, 23.6 mm SL, Mato Grosso, Boiadeiro Creek; LIRP 4435, 14 of 16, 20.7–26.6 mm SL, 2 c&s, 23.0–23.7 mm SL, Mato Grosso, Sapo Creek. *Aspidoras virgulatus*: MBML 6750, 6 of 9, 23.5–30.2 mm SL, 2 c&s, 32.2–33.0 mm SL, Bahia, Manoelzinho Stream.
